# Induction of Tenogenic Differentiation Mediated by Extracellular Tendon Matrix and Short-Term Cyclic Stretching

**DOI:** 10.1155/2016/7342379

**Published:** 2016-08-18

**Authors:** Janina Burk, Amelie Plenge, Walter Brehm, Sandra Heller, Bastian Pfeiffer, Cornelia Kasper

**Affiliations:** ^1^Translational Centre for Regenerative Medicine, University of Leipzig, Philipp-Rosenthal-Strasse 55, 04103 Leipzig, Germany; ^2^Saxon Incubator for Clinical Translation, University of Leipzig, Philipp-Rosenthal-Strasse 55, 04103 Leipzig, Germany; ^3^Institute of Veterinary Physiology, University of Leipzig, An den Tierkliniken 7, 04103 Leipzig, Germany; ^4^Large Animal Clinic for Surgery, University of Leipzig, An den Tierkliniken 21, 04103 Leipzig, Germany; ^5^Department of Pathology and Laboratory Medicine, Tulane University, 1430 Tulane Avenue, New Orleans, LA 70112, USA; ^6^Department of Biotechnology, University of Natural Resources and Life Sciences (BOKU), Muthgasse 18, 1190 Vienna, Austria

## Abstract

Tendon and ligament pathologies are still a therapeutic challenge, due to the difficulty in restoring the complex extracellular matrix architecture and biomechanical strength. While progress is being made in cell-based therapies and tissue engineering approaches, comprehensive understanding of the fate of progenitor cells in tendon healing is still lacking. The aim of this study was to investigate the effect of decellularized tendon matrix and moderate cyclic stretching as natural stimuli which could potentially direct tenogenic fate. Equine adipose-derived mesenchymal stromal cells (MSC) were seeded on decellularized tendon matrix scaffolds. Mechanical stimulation was applied in a custom-made cyclic strain bioreactor. Assessment was performed 4 h, 8 h, and 24 h following mechanical stimulation. Scaffold culture induced cell alignment and changes in expression of tendon-related genes, although cell viability was decreased compared to monolayer culture. Short mechanical stimulation periods enhanced most of the scaffold-induced effects. Collagen 1A2 expression levels were decreased, while collagen 3A1 and decorin levels were increased. Tenascin-C and scleraxis expression showed an initial decrease but had increased 24 h after stimulation. The results obtained suggest that decellularized tendon matrix, supported by cyclic stretching, can induce tenogenic differentiation and the synthesis of tendon components important for matrix remodeling.

## 1. Introduction

Treatment of tendon and ligament pathologies is still a major challenge in orthopedics. In many cases, the original functionality cannot be restored by conventional therapies. Tendons and ligaments are characterized by low cellularity and highly structured extracellular matrix displaying extraordinary mechanical strength and elasticity [1, 2]. Reparative processes within these tissues include the formation of hypercellular scar tissue lacking physiological matrix composition and structure, which results in inferior biomechanical properties and high risk of reinjury [1, 3]. Considerable progress has been made in treatment strategies based on progenitor cell application and tissue engineering techniques. Here, improvement of tissue (re)organization is a major aim in order to achieve mechanical strength and elasticity [2].

Studies in the equine large animal model indicate that local application of multipotent mesenchymal stromal cells (MSC) for treatment of tendon disease supports the reorganization of physiological tissue architecture [4–6] and improves tendon biomechanical properties [6]. However, the mechanisms mediating these improvements are not yet completely understood. Replacement of tenocytes by the applied MSC after their tenogenic differentiation could be part of the complex processes, together with the modulation of local tenocyte and leukocyte activity [6]. Although tenocyte replacement itself is unlikely to be the major contribution to tendon regeneration [7], tenogenic differentiation being accompanied by production of important tendon matrix molecules could be a crucial mechanism to support matrix reorganization. While collagens, namely, collagen 1A2, are the most important tendon matrix components on a quantity basis, further molecules are of importance, particularly with respect to the matrix architecture [2]. For example, decorin and tenascin-C are involved in collagen fibrillogenesis and are expressed even by unstimulated, cultured MSC [8].

Aiming to understand and optimize regenerative tendon therapy and tendon tissue engineering strategies, it is still crucial to improve the understanding of stimuli for the tenogenic pathway and tendon extracellular matrix remodeling. Tenogenic differentiation has been investigated in vitro using a wide variety of approaches. These include supplementation with growth factors [9–11] or transfection of the cells with the tenogenic transcription factor scleraxis [12, 13] as well as the application of different scaffold biomaterials or mechanical stimulation of the cells. Although several authors reported promising results, the fact that tenogenic differentiation is being approached using these diverse stimuli underlines that there is not yet a commonly accepted understanding of the pathway.

For scaffold materials, it has been shown that, on the one hand, topographic cues in artificially assembled scaffolds can direct lineage-specific differentiation [14, 15]. On the other hand, pulverized natural tendon components also induced tendon marker upregulation [16, 17], although other studies reported conflicting results [18, 19]. Furthermore, osteogenesis markers were expressed at lower levels when pulverized tendon matrix was added to constructs [17]. Consequently, decellularized natural tendon tissue would perfectly combine topographic and matrix component stimuli for tenogenic differentiation. According to this hypothesis, tendon progenitor cells cultured on decellularized tendon matrices showed a higher expression of tenogenic transcription factors compared to cells cultured on bone or dermis matrices [20].

Mechanical stimulation in the form of static tension or cyclic axial stimulation has led to encouraging results in most studies. In comparison to static tension, cyclic strain facilitated cell alignment and upregulation of tendon matrix molecules such as collagen 1, collagen 3, and tenascin-C and tendon differentiation markers such as scleraxis [21–25]. Low or moderate mechanical stimulation regimes further improved construct biomechanical properties [26]. However, cyclic axial strain can also upregulate osteogenic differentiation markers [27], apparently being dependent on the stimulation regime applied [28].

Based on the knowledge existing when the current study was initiated, we concluded that culture on decellularized tendon scaffolds combined with mechanical stimulation would be a crucial step to further investigate stimuli for tenogenic differentiation. After optimization of a decellularization protocol for large tendons [29] and preliminary experiments using a cyclic strain bioreactor [30] for stimulation of monolayer cultures, we developed a bioreactor suitable for applying cyclic strain to seeded decellularized tendon scaffolds [31]. The aim of this study was to gain a better understanding of stimuli leading to the synthesis of important tendon matrix components, hypothesizing that combined stimulation by the scaffold and cyclic strain would regulate their expression.

## 2. Materials and Methods

### 2.1. Mesenchymal Stromal Cell Recovery

Subcutaneous adipose tissue was recovered from 6 healthy horses aged 1–5 years that were euthanized due to reasons unrelated to this study. The tissue was minced and digested in collagenase I solution (0.8 mg/mL; Life Technologies, Karlsruhe, Germany) at 37°C for 4 h and released cells were seeded in tissue culture flasks using Dulbecco's modified Eagle medium with 1 g glucose/L (Life Technologies) supplemented with 10% fetal bovine serum (Life Technologies), 0.05 mg/mL (0.1%) gentamycin (PAA Laboratories, Coelbe, Germany), 100 I.U./mL penicillin, and 0.1 mg/mL streptomycin (1% penicillin-streptomycin; Life Technologies) as standard culture medium. Cells were incubated in a humidified atmosphere with 5% CO_2_ at 37°C and medium was changed twice weekly. Passage 1 cells were cryopreserved and then further expanded under standard conditions prior to being used for the experiments in passage 3. MSC characteristics of equine adipose-derived cells have previously been described by our group [32, 33].

### 2.2. Tendon Scaffold Preparation

Superficial digital flexor tendons were recovered from equine cadaver limbs obtained from an abattoir. They were washed in phosphate buffered saline (Biochrom, Berlin, Germany) supplemented with 2% penicillin-streptomycin and 0.1% gentamycin at 4°C overnight and stored at −80°C until further processing. Next, tendons were pooled and decellularized as described before [29]. Briefly, they were subjected to 5 freeze-thaw cycles before incubation in aqua dest for 48 h, followed by incubation in 1% Triton-X 100 (Carl Roth, Karlsruhe, Germany) for 48 h and washing steps. Subsequent to decellularization, tendons were cut into scaffolds (10 or 8 cm length, 1 cm width, and 2 mm thickness). Scaffolds were stored at −80°C until being used for cell culture.

### 2.3. Cyclic Strain Bioreactor

**Figure 1:**
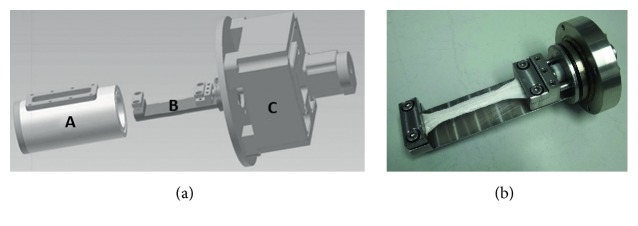
(a) Schematic drawing of the custom-made cyclic strain bioreactor; A: medium chamber; B: stretching device; C: motor unit. (b) Photograph of the stretching device with fixed tendon scaffold.

To enable cyclic stretching of the tendon scaffolds, a custom-made cyclic strain bioreactor was developed based on data obtained from a preliminary biomechanical assessment of the decellularized tendon scaffolds. With relevance to the choice of the motor, these data had shown that loads approximating 500 N were necessary to stretch the scaffolds as required [31]. The stretching device is located in a cylindrical medium chamber and includes clamps to fix a 10 cm long tendon scaffold at both ends while 8 cm scaffold length remains free to be stretched. One of the clamps is freely moveable along the tendon axis and coupled with a 1 kN motor in order to apply uniaxial cyclic strain (Figure 1).

### 2.4. Stimulation Experiments

To analyze the impact of tendon extracellular matrix and cyclic stretching on the MSC, experiments were carried out with the following experimental groups: static scaffold culture (static), dynamic scaffold culture according to stimulation regime I (stim I) or stimulation regime II (stim II), and monolayer control cultured on standard plastic culture dishes.

MSC were homogeneously distributed on the surface of prewarmed scaffolds (10^6^ cells/cm^2^) and allowed to attach for 6 h until standard culture medium was added. Seeded scaffolds were incubated at standard conditions for 3 days.

Dynamic groups were then subjected to cyclic stretching intervals with 2% strain at a frequency of 1 Hz (stim I: 15 min stretching, 15 min rest, and 30 min stretching; stim II: 15 min stretching, 15 min rest, 30 min stretching, 30 min rest, and 60 min stretching). Seeded scaffolds were analyzed 4, 8, and 24 h after the last stretching interval.

Static scaffold cultures and monolayer cultures were prepared and analyzed at the same corresponding time points. Each experiment was carried out separately with the MSC from each donor (*n* = 6) and in duplicate.

### 2.5. Histology

To evaluate cell viability as well as cell morphology, alignment and distribution on the scaffold surface, central, and peripheral regions of the freshly harvested samples were subjected to LIVE/DEAD staining using the respective kit (Life Technologies) according to the manufacturer's instructions.

To further evaluate cell distribution and integration into the scaffolds as well as collagen matrix production, paraffin sections were prepared from central and peripheral parts of the samples which were subjected to hematoxylin and eosin staining and immunohistochemical staining of procollagens.

Immunostaining was performed using goat anti-human procollagen 1A1 polyclonal antibodies or mouse anti-human procollagen 3A1 monoclonal antibodies (A-17 or B-4; Santa Cruz Biotechnology, Heidelberg, Germany) combined with Immunocruz*™* ABC staining systems (Santa Cruz Biotechnology) containing donkey anti-goat or goat anti-mouse secondary antibodies. Briefly, sections were deparaffinized, incubated with methanol and 0.005% H_2_O_2_ for 30 min, and washed. Slides were then incubated with the respective primary antibodies (1 : 50) at 4°C overnight. After further washing steps, they were incubated with the secondary antibodies (1 : 50) for 30 min, followed by detection steps detailed in the manufacturer's instructions. Counterstaining was performed with hematoxylin. Isotype controls as well as negative controls omitting the primary antibodies were prepared accordingly.

LIVE/DEAD staining was evaluated by 1 observer immediately after processing. All stained paraffin sections were evaluated by 2 independent observers ignorant of the sample group. The score systems used for semiquantitative assessment are shown in Table 1.

### 2.6. Real-Time PCR

Gene expression of the tendon extracellular matrix components and tendon differentiation markers collagen 1A2, collagen 3A1, decorin, tenascin-C, and scleraxis was analyzed by real-time PCR. Collagen 2A1 and osteopontin (i.e., secreted phosphoprotein 1) expression was additionally assessed to detect potential induction of chondrogenesis or osteogenesis. GAPDH and ACTB served as housekeeping genes. Primer sequences are given in Table 2.

Tendon constructs were sliced with a cryomicrotome (CM 3050 S; Leica Microsystems, Wetzlar, Germany) and incubated in homogenization buffer (15 mM HEPES, 2.5 mM KCl, 68.5 mM NaCl, 450 *μ*M Na2HPO4, 17.5 mM EDTA, and 27.5 mM glucose at pH 7) containing 100 *μ*g/mL proteinase K (Life Technologies) at 55°C for 60 min. After homogenization, total RNA was purified with phenol/chloroform extraction and isopropanol precipitation. Total RNA of control MSC was isolated using the RNeasy® Mini Kit (Qiagen, Hilden, Germany) according to instructions of manufacturers (protocol version 09/2010). DNase-treated RNA was reverse transcribed using the RevertAid H Minus First Strand cDNA Synthesis Kit (Thermo Fisher Scientific, Nidderau, Germany) with oligo-dT18 primers as described by the manufacturers. Relative quantification of cDNA was performed with an Applied Biosystems® 7500 Real-Time PCR System (Life Technologies) and SYBR® green as double-strand DNA-specific dye (iQ*™*SYBR Green Supermix, Bio-Rad Laboratories, Munich, Germany).

Relative gene expression ratios were calculated according to the Pfaffl method [34] and normalized to those obtained from the respective monolayer controls. Data are presented as “fold change” increase (FC_i_ = (ratio_treated_/ratio_control_) − 1) or decrease (FC_d_ = 1/(ratio_treated_/ratio_control_) − 1).

### 2.7. Statistical Analysis

Mean values of duplicates and different sample regions (central or peripheral) were used for the final statistical analysis. Using SPSS Statistics 22 (IBM Deutschland GmbH, Ehningen, Germany), Friedman tests and Wilcoxon signed-rank tests were performed to analyze differences between the experimental groups. The level of significance was set at *p* = 0.05.

## 3. Results

### 3.1. Cell Viability

**Figure 2:**
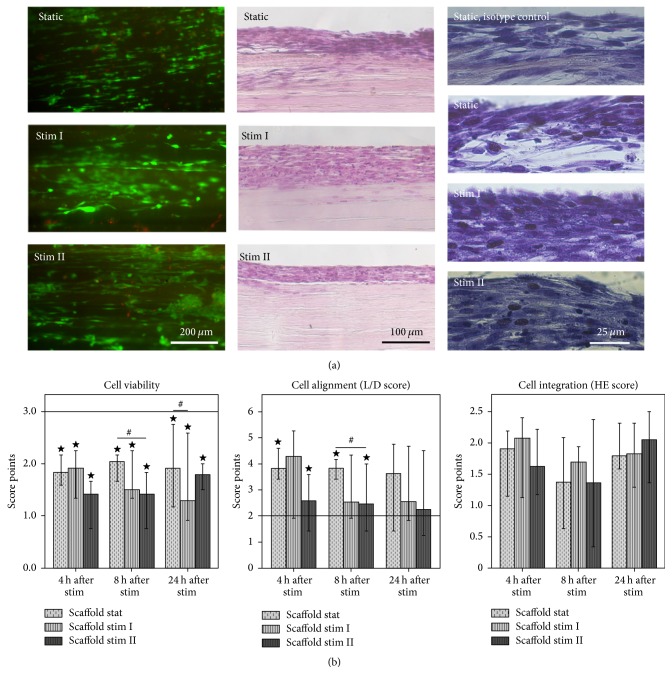
(a) Microphotographs of LIVE/DEAD® staining of seeded scaffold samples (viable cells shown in green, dead cells in red; left), hematoxylin and eosin staining of paraffin sections (middle), and procollagen 3A1 immunostaining of paraffin sections (positive staining in dark brown; right), 24 h after mechanical stimulation. (b) Score points for cell viability (left) or cell morphology, alignment, and distribution on the scaffold surface (L/D score; middle), obtained by evaluation of LIVE/DEAD stained samples, the horizontal lines representing the monolayer controls, and score points for cell integration and cell layer integrity (HE score, right), obtained by evaluation of hematoxylin and eosin stained sections; bars represent the median values and error bars the 95% confidence interval; ⋆ marks significant differences compared to the monolayer control (*p* < 0.05); # means significant differences between the sample groups indicated (*p* < 0.05); stat: static; stim: mechanical stimulation.

LIVE/DEAD staining revealed that scaffold culture decreased the percentage of living cells compared to the monolayer controls (*p* < 0.05). In most samples cultured on scaffolds, 50–75% of MSC were vital, corresponding to 2 score points, whereas, in all monolayer samples, more than 75% of MSC were vital, corresponding to 3 score points. This difference was observed at all analysis time points (4, 8, and 24 h), that is, after a total of 3 to 4 days of scaffold or monolayer culture. Moreover, mechanical stimulation tended to further affect cellular viability, but this was only significant for the stim II group at 8 h and for the stim I group at 24 h (*p* < 0.05 compared to static scaffold culture) (Figure 2).

### 3.2. Cell Alignment and Integration

Evaluation of cell morphology and orientation on the scaffold surface using the LIVE/DEAD stained samples showed that scaffold culture strongly promoted parallel alignment and the appearance of more slender, elongated cells. This led to significant differences between the score points of scaffold groups compared to the monolayer controls (*p* < 0.05 for the static and stim II groups at 4 h and 8 h), although the other parameters included in the scoring did not differ. Mechanical stimulation appeared to decrease the scaffold-induced cell alignment, which became most evident comparing the score points obtained in the static and stim II groups at 8 h (*p* < 0.05), but cells remained still more aligned than in the monolayer controls. In some cases, partial cell detachment was observed after stretching mostly in the stim II group (Figure 2).

Hematoxylin and eosin staining of longitudinal sections showed that, in most scaffolds, the major part of MSC was located in the cell layer on the scaffold surface, with few cells starting to integrate into the tendon matrix. While there was a tendency that short stretching (stim I) had promoted cell integration at 4 h and 8 h, no significant differences between groups were found regarding the morphology of the cell layers on the scaffolds (Figure 2).

### 3.3. Tendon Marker Expression

**Figure 3:**
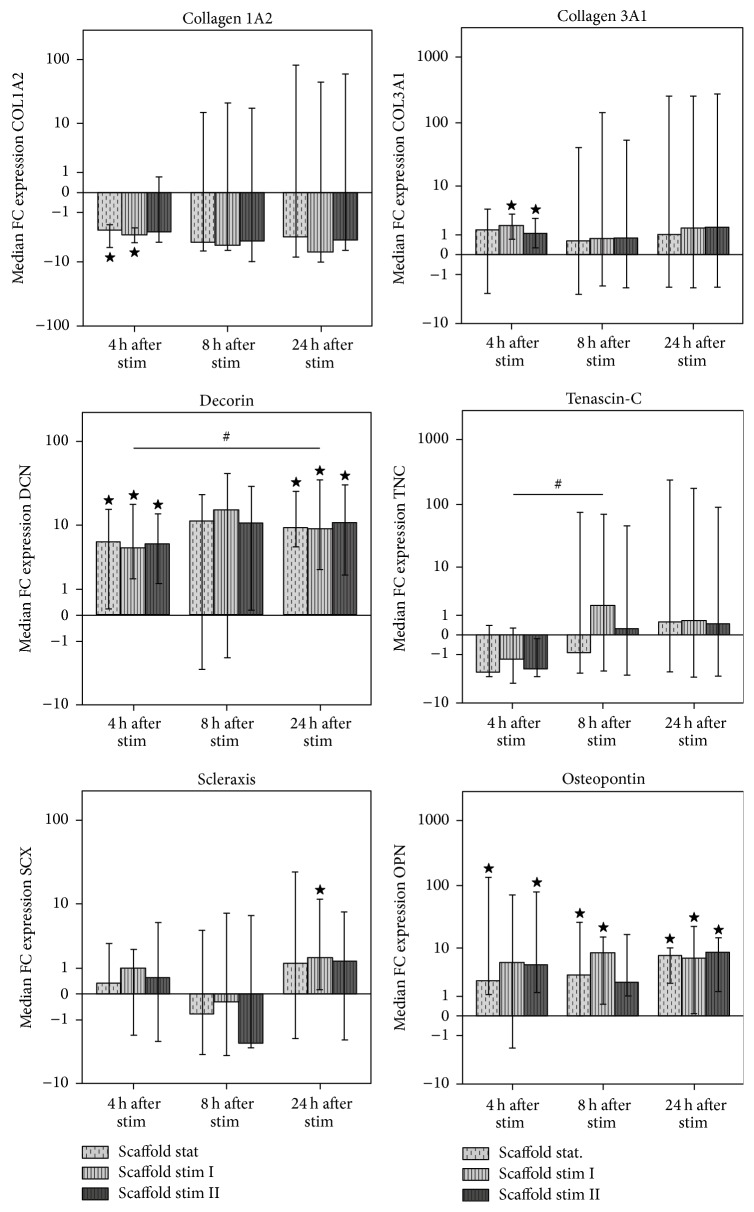
Gene expression levels of tendon markers and osteopontin, presented as “fold change” (FC) to the monolayer controls which are depicted by the horizontal line at 0. Bars represent the median values and error bars the 95% confidence interval; ⋆ marks significant differences compared to the monolayer control (*p* < 0.05); # means significant differences between the sample groups indicated (*p* < 0.05); stat: static; stim: mechanical stimulation.

Real-time PCR revealed that collagen 1A2 expression was downregulated after scaffold culture compared to the monolayer controls at all time points, which was significant for the static and stim I groups at 4 h (*p* < 0.05). This effect was enhanced by mechanical stimulation, particularly in the stim I group at 24 h, but this difference was not significant (Figure 3). Based on the PCR results, we did not expect significant immunohistochemical procollagen 1 staining in the seeded scaffolds. Therefore, staining was only performed exemplarily using the samples harvested at 8 h, which were all negative except for 1 weakly positive sample in the stim I group.

Contrary to collagen 1A2, collagen 3A1 levels were higher after scaffold culture compared to the monolayer controls at all time points, which was more pronounced when scaffolds had been subjected to mechanical stimulation (*p* < 0.05 for the stim I and stim II groups at 4 h). However, variations in collagen 3A1 expression were relatively high (Figure 3). Immunohistochemical staining of procollagen 3 revealed no further distinctive differences. While most samples showed no or very weak staining, individual samples stained positive (3/6 in the stim I group at 4 h; 3/6 in the static group at 24 h; 1/6 or 2/6 in all other groups) but with no recognizable pattern with respect to staining intensity and stimulation regime (Figure 2).

Distinctive changes in gene expression were found for decorin, with elevated expression levels in all scaffold cultures (static, stim I, and stim II) compared to monolayer controls at all time points, irrespective of mechanical stimulation (*p* < 0.05 at 4 and 24 h). Moreover, decorin expression levels in all scaffold groups increased further over time. At 8 h, although median expression levels were already higher compared to 4 h, high variations within the groups were evident. However, the increase over time became manifest in the stim I group when comparing decorin levels at 4 h and 24 h (*p* < 0.05) (Figure 3).

No significant differences were found between tenascin-C gene expression levels in different groups. However, expression in the scaffold groups increased over time. While at 4 h tenascin-C expression in all scaffold culture groups was lower than in the monolayer controls, it was slightly higher in all scaffold culture groups at 24 h, with the fastest increase in the stim I group as observed at 8 h (*p* < 0.05 compared to 4 h) (Figure 3).

Scleraxis expression also showed differential patterns over time. At 4 h, expression levels in scaffold culture groups were similar but slightly higher compared to the monolayer controls but had decreased to lower levels than the monolayer controls at 8 h. However, at 24 h, upregulation could be observed, the scaffold culture groups expressing scleraxis at higher levels than the monolayer controls. Among the scaffold culture groups, the stim I group showed the highest scleraxis expression at all time points, which was significant compared to monolayer cells at 24 h (*p* < 0.05) (Figure 3).

Collagen 2A1 was not expressed at detectable levels, neither in monolayer controls nor in any scaffold group. However, osteopontin was not only detectable in all sample groups but upregulated during scaffold culture with increasing expression levels over time. As observed at 8 h, the fastest increase was found in the stim I group, but values were again similar for all scaffold groups at 24 h. Differences in osteopontin expression compared to monolayer cells were significant for the static and stim II groups at 4 h, for the static and stim I groups at 8 h and for all scaffold culture groups at 24 h (*p* < 0.05) (Figure 3).

## 4. Discussion

In the current study, we investigated the effects of extracellular tendon matrix and cyclic stretching, combined in a new in vitro model system, on the tenogenic fate of adipose-derived MSC. The data obtained indicate that these crucial stimuli promote tenogenic induction and tendon matrix synthesis by the MSC within a short period of time.

Adipose tissue was used for MSC recovery as it is commonly considered as a highly promising progenitor cell source [35] and adipose-derived MSC were previously found to display higher basic expression of tendon matrix components than MSC from other sources [8]. Equine tissues were used as the horse is the large model animal species in which most experiences exist regarding tendon pathophysiology [1] as well as MSC characteristics and MSC-based tendon therapies [36]. Furthermore, all experiments were performed with MSC from each of the 6 donor animals, without pooling the cells. This is likely to be the reason for the relatively high variations in gene expression found in the different experimental groups. While, to some extent, these variations impede the significance of results, they represent the natural biological variability which is existent in human beings as well.

The decellularized tendon matrices were chosen as scaffolds as they best represent natural tendon components and architecture, as detailed above. While this approach led to interesting data, it should be acknowledged that synthetic materials could be advantageous regarding reproducibility of scaffold design and data obtained using scaffolds. Furthermore, with regard to the further development of the in vitro model, systematic use of synthetic scaffolds reflecting selected aspects of the extracellular tendon matrix could be of high relevance to identify the most relevant stimuli. Comparative approaches using extracellular matrices at the same time as synthetic scaffolds could therefore be useful to gain more insight.

The mechanical stimulation regimes were chosen based on our own preliminary experiments, previous publications, and the conditions encountered in vivo during rehabilitation. The applied frequency of 1 Hz roughly matches the frequency of natural movements and was used in several previous studies [22–24, 28, 37]. In contrast, the magnitude of strain applied differs considerably between studies reported previously, ranging from 1% [11] to 10% [21, 22, 26, 37]. When different strain magnitudes were applied in the same study, higher magnitudes led to higher increases in tendon marker expression [23, 25, 28]. On the other hand, moderate strain magnitudes of 2.5% improved the biomechanical properties of the construct better than higher strains [26] and were more feasible to apply to the scaffolds used in the current study without provoking damage. Moreover, it is known that, in vivo, the equine superficial digital flexor tendon, which is the tendon best comparable to the human Achilles tendon, experiences strains of roughly 2% at the walk [38]. Based on that, we chose to apply strains of 2% magnitude in our experiments. Furthermore, the duration of mechanical stimulation and its potential repetition on subsequent days are variables likely to influence the results, which were set in diverse ways in different previous studies [10, 21–23, 28, 39]. In preliminary experiments, we found that stretching for 60 min, applied without previous shorter adaptation periods, led to high rates of cell death. Stretching with shorter initial and increasing subsequent intervals showed less impact on cellular viability, although cell survival was still decreased compared to the static cultures. Therefore, two different stimulation regimes with increasing stress/rest periods were applied in the current study.

With respect to cell viability, it should be acknowledged that not only mechanical stimulation but also the scaffold culture showed some negative impact. It is possible that not all seeded cells were capable of adapting to the new environment quickly. In this case, after the first adaptation period, the cells are likely to remain viable over longer periods of time. Nevertheless, it remains to be excluded that matrix alterations due to decellularization or residual decellularization agents are responsible for cytotoxic effects [40]. Yet, extensive washing steps were included in the decellularization protocol used in this study to minimize residues, and our previous work demonstrated good cytocompatibility of the current scaffolds over 14 days [29].

We further decided to apply the stimulation regimes only once and to perform the assessment over the first 24 h after stretching. This enabled us to monitor early changes in gene expression shortly after potential induction of tenogenic differentiation when combining mechanical and tendon matrix stimuli in this setting for the first time. Contrary to the current approach, longer or repeated stretching for 1, 2, or 3 weeks was applied in most other studies focusing on tenogenic differentiation [10, 11, 23–26, 39], 24 h after stimulation being the first assessment time point in only few of these [11, 24].

Using the approach described, we found significant scaffold-induced changes in cell alignment as well as in gene expression of tendon markers. With regard to the latter, there was a uniform tendency that short mechanical stimulation (stim I) augmented the effects mediated by the scaffolds, which could be observed consistently for most genes investigated. Furthermore, particularly for scleraxis and tenascin-C, highly interesting time-dependent changes in expression levels were evident during the first 24 h after stretching.

Gene expression of collagens remained relatively constant at all investigated time points, collagen 1A2 being downregulated and collagen 3A1 being upregulated in all scaffold culture groups. Furthermore, collagen 3A1 gene expression levels were higher than collagen 1A2 levels in all groups and at all time points (data not shown). Immunostaining of procollagens revealed no distinctive difference between groups on protein level, potentially due to the early assessment time points, but reflected higher expression of collagen 3 compared to collagen 1. On the one hand, scaffold-induced downregulation of collagen 1 might be due to the high amounts of the protein being present in the scaffold. However, it was shown that collagen 1 levels were increased after 7 days of culture in the presence of tendon matrix [16]. Collagen 1 was also upregulated by cyclic stretching at day 7, but, corresponding to the present findings, it was initially downregulated, which was evident at day 1 and day 3 [24]. In the same study, collagen 3 expression showed a similar tendency but increased faster [24]. Thus, the present findings are supported by this previous study and might actually reflect the situation of the early tendon healing phase in vivo, at which collagen 3 is synthesized first to replace damaged tendon matrix, followed by collagen 1 [1].

The most dominant changes in gene expression were observed for decorin, which was strongly increased in all scaffold groups. These changes in gene expression appeared to be mediated by the scaffold, with no clear additional effect of stretching. Interestingly, no changes in decorin expression were detected after culture in poly-L-lactide or collagen gel scaffolds, with or without mechanical stimulation [23, 24]. This suggests that the combination of topographical and biochemical cues provided by natural tendon matrix might be a key component to induce decorin expression. Furthermore, the fact that decorin was upregulated in the current experimental setting, imitating natural tendon environment, supports the hypothesis that decorin may play a crucial role in MSC-based tendon therapy, potentially contributing to an improved matrix architecture.

Tenascin-C and scleraxis showed different gene expression levels at different time points, illustrating their regulation over time. Interestingly, following initial decreases, observed at 4 h for tenascin-C and at 8 h for scleraxis, increases in the expression of both genes were evident at 24 h after stretching, apparently being induced by the scaffold stimuli and enhanced by the mechanical stimuli. Corresponding to these results, tenascin-C expression was found to be increased by mechanical stimulation although not as early as on day 1 [21–23]. No effect on tenascin-C expression was revealed when using pulverized tendon matrix [16], potentially due to the lack of topographical cues. Scleraxis was upregulated when using collagen gel scaffolds and further enhanced by cyclic loading, which supports the results of the current study [41]. Similarly, it was shown that scleraxis was upregulated by collagen gel scaffold culture and that cyclic loading supported maintenance of scleraxis expression for 7 days [24]. However, in the same study, scleraxis expression was not further increased by mechanical stimulation [24]. Regulation of scleraxis expression shortly after mechanical stimulation was not described so far. The present results demonstrate that even short and moderate mechanical stimulation combined with the use of appropriate scaffold material induces scleraxis and tenascin-C upregulation within 24 h. This indicates a rapid induction of tenogenic differentiation, which would potentially be followed by further regulation of extracellular matrix synthesis.

Collagen 2A1 gene expression could not be detected in any of the samples, but osteopontin expression was increased in all scaffold groups. Increased expression of osteogenesis-related markers induced by cyclic axial stretching was described before [27, 28, 30]. The present finding indicates that the use of tendon matrix as scaffold does not prevent this effect; hence, osteogenic differentiation of applied cells could still be a serious risk in tendon cell therapies. Nevertheless, while these in vitro data should not be neglected, no evidence of calcifications within MSC-treated tendons was found in vivo in equine probands [42].

Based on the knowledge on the early period after tenogenic induction obtained so far in our model system, further studies including repeated application of mechanical stimulation and longer incubation periods would be indicated. Intriguingly, studies using an approach very similar to the current one, combining cell culture on decellularized tendon scaffolds and mechanical stimulation, were published recently [39, 43]. Here, mechanical stimulation was performed over 8 or 10 days until samples were harvested; hence, the results of the present study add to these published data very well. Youngstrom et al. found increased gene expression levels of scleraxis, collagen 1, and decorin but decreased expression of collagen 3 when using a stimulation regime with 3% strain, being accompanied by improved biomechanical properties [39]. Considering Youngstrom et al.'s and our findings at the same time, it can be assumed that scleraxis and decorin expression levels not only are elevated very early but can be maintained in the model system used. In contrast, different results were obtained regarding collagen expression, most likely due to the longer incubation and later assessment in Youngstrom et al.'s studies. Consequently, the respective changes in collagen expression profiles could be expected during the first week of tenogenic differentiation.

## 5. Conclusions

The data obtained in the current study indicate that extracellular tendon matrix and moderate cyclic axial stretching promote tenogenic differentiation and tendon matrix synthesis by MSC. Furthermore, changes in cell fate appeared to be predominantly directed by the decellularized tendon scaffold and were evident within a short period of time.

The model system used imitates crucial aspects of the conditions encountered by MSC after their intratendinous application. Therefore, the current results support the hypothesis that the beneficial effect of MSC observed in equine tendon therapy could be partially mediated by cell replacement and matrix remodeling. However, disease modeling should be improved on the basis of the existing model system in order to understand how progenitor cell fate is directed under pathological conditions.

## Figures and Tables

**(a):** 

Viability score	
Description	Score points
<10% vital cells	0
10–50% vital cells	1
>50–75% vital cells	2
>75% vital cells	3

**(b):** 

L/D score					
Cell morphology		Cell alignment		Cell distribution	
Description	Score points	Description	Score points	Description	Score points
Predominantly rounded	0	Predominantly random	0	Focal	0
Predominantly polygonal or equal numbers rounded and elongated	1	Equal numbers random and parallel	1	Multifocal	1
Predominantly elongated	2	Predominantly parallel	2	Homogeneous	2

**(c):** 

HE score			
Cell integration		Cell layer integrity	
Description	Score points	Description	Score points
None	0	Single cells only	0
Integration of single cells	1	Nonhomogeneous cell layer	1
Integration of >50% of cells	2	Homogeneous cell layer	2

**Table 2:** Primer sequences.

Equine gene	Forward primer	Reverse primer	Accession number	PCR product in bp
ACTB	ATCCACGAAACTACCTTCAAC	CGCAATGATCTTGATCTTCATC	NM_001081838.1	174
GAPDH	TGGAGAAAGCTGCCAAATACG	GGCCTTTCTCCTTCTCTTGC	NM_001163856.1	309
Collagen 1A2	CAACCGGAGATAGAGGACCA	CAGGTCCTTGGAAACCTTGA	XM_001492939.1	243
Collagen 3A1	AGGGGACCTGGTTACTGCTT	TCTCTGGGTTGGGACAGTCT	XM_001917620.2	216
Decorin	ACCCACTGAAGAGCTCAGGA	GCCATTGTCAACAGCAGAGA	NM_001081925.2	239
Tenascin-C	TCACATCCAGGTGCTTATTCC	CTAGAGTGTCTCACTATCAGG	XM_001916622.2	163
Scleraxis	TACCTGGGTTTTCTTCTGGTCACT	TATCAAAGACACAAGATGCCAGC	NM_001105150.1	51
Collagen 2A1	ATTGTAGGACCCAAAGGACC	CAGCAAAGTTTCCACCAAGG	NM_001081764.1	199
Osteopontin	TGAAGACCAGTATCCTGATGC	GCTGACTTGTTTCCTGACTG	XM_001496152.2	158
